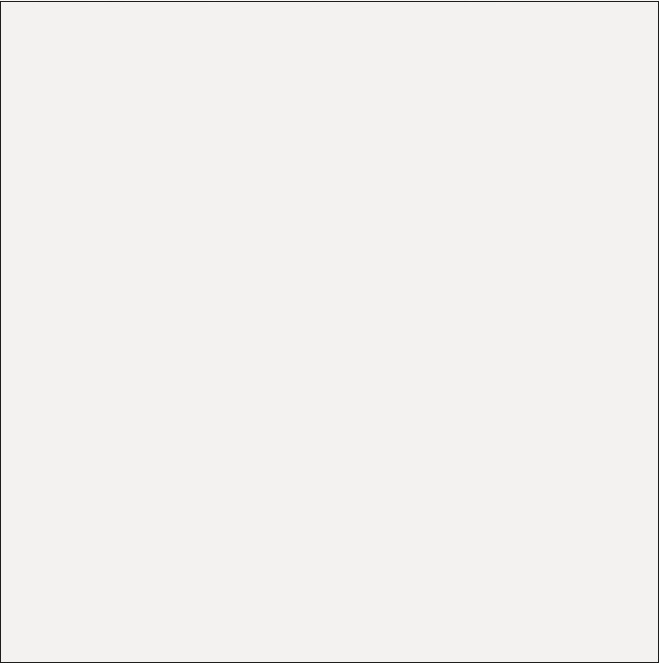# Test your knowledge and understanding

**Published:** 2014

This page is designed to test your understanding of the concepts covered in this issue and to give you an opportunity to reflect on what you have learnt. The multiple true/false questions were produced in collaboration with the International Council of Ophthalmology (ICO). Please visit www.cehjournal.org to complete these questions online.

**Table:** 

**1.**	**Think about the barriers to cataract services**	**True**	**False**
**a**	Cataract surgical coverage is usually higher in men than in women.	□	□
**b**	Most patients who are blind from cataract are willing to come for surgery no matter how much it costs.	□	□
**c**	Traditional beliefs, e.g. about going blind when the hair turns white, are no longer important.	□	□
**d**	A person who has had successful cataract surgery can be helpful in persuading others to come for surgery.	□	□
**2.**	**Think about the costs and quality of cataract services**	**True**	**False**
**a**	If a hospital does a small number of operations and has a waiting list it means there is a need to increase patient demand for services.	□	□
**b**	All patients should have presenting visual acuity better than 6/18 one month after cataract surgery.	□	□
**c**	Offering tiered fees for cataract services is one way in which paying patients can subsidise the costs for poor patients.	□	□
**d**	Accurate biometry is important in providing good quality cataract surgery.	□	□

## ANSWERS

**1.a. True.** Women tend to face more barriers to accessing cataract services than men. **b. False.** The cost of surgery is an important barrier for poor people. **c. False.** Traditional beliefs remain an important reason why people do not come for surgery. **d. True.** For many people, meeting someone who has undergone surgery and who has had a good outcome will boost their confidence and motivate them to come for surgery.
**2. a. False.** If there is a waiting list this needs to be dealt with before creating further demand. **b. False.** This is not possible because some patients will have ocular co-morbidities such as glaucoma or retinal disease and some will suffer from complications during surgery. However, the aim should be that as many patients as possible achieve this vision post-operatively, and every effort should be made to make this happen. The causes of poor visual outcome will only be known if information about post-operative visual acuity is collected routinely. **c. True.** This model involves charging extra for non-clinical services like a private room and to use the profit to subsidise the cost for poor patients. **d. True.** Accurate biometry means there is no – or less – refractive error after surgery and so the visual acuity results are usually better.

**Time to reflect**

1. How relevant to your day-to-day work was the material covered in this issue of the *Community Eye Health Journal?* **Extremely relevant, relevant, neither relevant nor irrelevant, irrelevant, extremely irrelevant** (circle as appropriate)
2. How much of what you read in this issue was new to you? Please give a percentage:
  **Figure:**
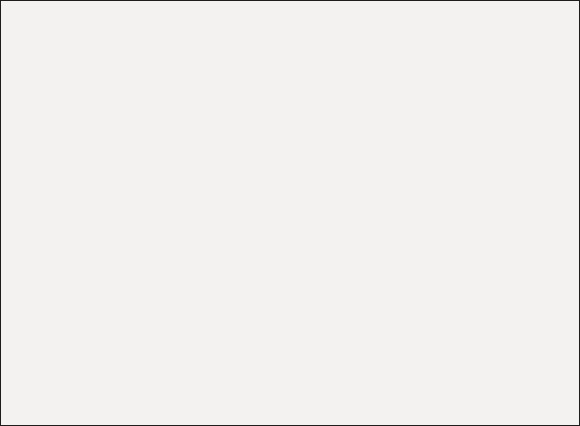
3. As a result of reading this issue, will you be changing your practice/teaching/leadership/policies/management? **Yes/No** (circle as appropriate)
4. If ‘Yes’, give examples of planned changes in the space provided, or in your own continued professional development (CPD) diary.
  **Figure:**